# Ontogeny of the Projections From the Dorsomedial Division of the Anterior Bed Nucleus of the Stria Terminalis to Hypothalamic Nuclei

**DOI:** 10.3389/fnins.2021.748186

**Published:** 2021-11-30

**Authors:** Marc Lanzillo, Manon Gervais, Sophie Croizier

**Affiliations:** Center for Integrative Genomics, University of Lausanne, Lausanne, Switzerland

**Keywords:** DiI-based tract tracing, ontogeny, bed nuclei of the stria terminalis, PVH, DMH, ARH, LHA

## Abstract

The bed nucleus of the stria terminalis (BNST) is a telencephalic structure well-connected to hypothalamic regions known to control goal-oriented behaviors such as feeding. In particular, we showed that the dorsomedial division of the anterior BNST innervate neurons of the paraventricular (PVH), dorsomedial (DMH), and arcuate (ARH) hypothalamic nuclei as well as the lateral hypothalamic area (LHA). While the anatomy of these projections has been characterized in mice, their ontogeny has not been studied. In this study, we used the DiI-based tract tracing approach to study the development of BNST projections innervating several hypothalamic areas including the PVH, DMH, ARH, and LHA. These results indicate that projections from the dorsomedial division of the anterior BNST to hypothalamic nuclei are immature at birth and substantially reach the PVH, DMH, and the LHA at P10. In the ARH, only sparse fibers are observed at P10, but their density increased markedly between P12 and P14. Collectively, these findings provide new insight into the ontogeny of hypothalamic circuits, and highlight the importance of considering the developmental context as a direct modulator in their proper formation.

## Introduction

The bed nucleus of the stria terminalis (BNST), a complex forebrain structure, has long been involved in stress- and anxiety-related behaviors (Walker et al., 2003). The BNST receives strong connections from the prefrontal cortex and the amygdala (Dong et al., 2001), and project in turn onto hypothalamic nuclei that mediate goal-oriented behaviors (Dong and Swanson, 2006; Barbier et al., 2021). Indeed, the dorsomedial part of the anterior BNST strongly projects onto the paraventricular (PVH), the dorsomedial (DMH), the arcuate (ARH) nuclei, and the lateral hypothalamic area (LHA). In particular, nociceptin-expressing neurons of the dorsomedial part of the BNST control feeding throughout arcuate AgRP neurons (Smith et al., 2019). In addition, corticotropin-releasing hormone (CRH)- and cholecystokinin (CCK)-expressing neurons of the dorsal part of the anterior BNST regulate aversive and appetitive behaviors via neurons of the LHA, including Orexin-expressing neurons (Jennings et al., 2013; Giardino et al., 2018). While we start to know more about the anatomy and the functional role of these projections, when the projections from the dorsomedial part of the anterior BNST to the aforementioned hypothalamic nuclei develop is still unknown.

Our work highlighted a postnatal formation of the neurocircuits arising from the dorsomedial part of the anterior BNST and innervating the PVH, DMH, ARH, and LHA. This study will serve as a developmental basis to further evaluate long-lasting functions supported by these neuronal networks, and how alteration of maternal environment can precipitate the onset of metabolic and anxiety-related disorders in adulthood.

## Materials and Methods

### Animals and Tissue Preparation

All experimental procedures were approved by the Veterinary Office of Canton de Vaud (#VD3193). Mice were housed in individual cages and maintained in a temperature-controlled room with a 12 h light/dark cycle and provided *ad libitum* access to water and standard laboratory chow (Kliba Nafag). C57Bl6/J neonatal mice (Charles River) were anesthetized with sodium pentobarbital (150 mg/kg) and perfused on P4, P8, P10, P12, P14, P16, P18, P20, and P22 with 4% paraformaldehyde solution in phosphate buffer (Applichem, PFA), pH 7.4. The brains were then embedded in 3% agarose, and sectioned from rostral to caudal to expose the anterior BNST without affecting the hypothalamic nuclei. Each brain block was stained with 2% Chicago Blue to visualize anatomic features. A small cristal of DiI (SantaCruz Biotechnology) was placed into the dorsomedial part of the anterior BNST under binocular loup (Zeiss). After implantation, brains were stored in 4% PFA for 6 weeks in the dark at 37°C. There were 70 μm-thick sections that were then cut through the hypothalamus using a vibratome. Sections were mounted on Superfrost slides and cover slipped with DAPI-Fluoromount (SouthernBiotech).

### Analysis

Confocal images of DiI-labeled fibers were observed and/or acquired through the hypothalamus of P4 (*n* = 4), P8 (*n* = 3–4), P10 (*n* = 3–4), P12 (*n* = 3–6), P14 (*n* = 4–6), P16 (*n* = 3–8), P18 (*n* = 3–7), P20 (*n* = 3–6), and P22 (*n* = 3–6) male mice using a Zeiss LSM 710 confocal microscope equipped with a 20X objective. Brains were collected from 2 to 4 independent litters. The following regions were analyzed: (1) the neuroendocrine PVH, (2) the autonomic posterior PVH, (3) dorsomedial (DMHdm) and ventrolateral (DMHvl) parts of the DMH, (4) dorsomedial (ARHdm) and ventromedial (ARHvm) parts of the ARH, and (5) LHA (perifornical area) at the level of the DMH and ARH. DAPI-labeled nuclei allowed a clear morphological delimitation. For the quantification of DiI-positive fiber density, each image was binarized and integrated intensity (total numbers of pixels) was then calculated for each image using Image J (NIH). For this purpose, regions of interest (ROI) with the following dimensions were centered over each region studied: 150 × 150 μm for the neuroendocrine PVH; 243 × 70 μm for the posterior PVH; 115 × 115 μm for the DMHdm and DMHvl; 90 × 90 μm for the ARHdm and ARHvm; and 100 × 100 μm for the LHA (perifornical area). These ROI have been chosen based on the size of the ARH in early ages, and to be able to compare fiber density in both ARHdm and ARHvm.

### Statistics

Statistical analyses were conducted using GraphPad Prism (version 9). Statistical significance was determined using one-way ANOVA and two-way ANOVA followed by Tukey’s *post hoc* test. *P* ≤ 0.05 was considered statistically significant. Graphs have been generated using Prism 9 software and all values were represented as mean ± SEM.

## Results

**FIGURE 1:**
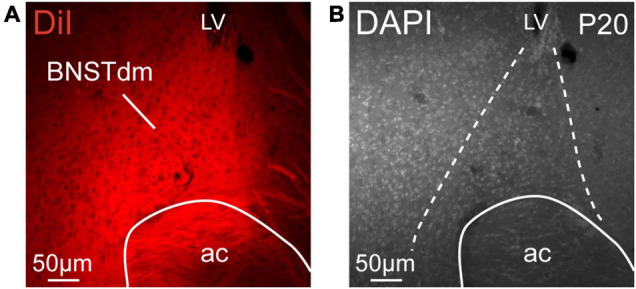
**(A)** Low magnification confocal image showing the distribution of DiI in the dorsomedial part of the anterior BNST (BNSTdm) of a P20 male mouse. **(B)** Low magnification image of a DAPI staining showing the cytoarchitectonic purposes of the BNSTdm. Dashed lines indicate the limit of the BNSTdm. ac, anterior commissure; LV, lateral. Scale bars are shown in the figure.

To examine the development of projections from the dorsomedial division of the anterior BNST to hypothalamic nuclei in male mice, we used the well-described DiI lipophilic fluorescent tracer. A representative injection site into the dorsomedial division of the anterior BNST is shown in Figure 1. Correct injection sites were obtained in 49 cases from P4 to P22 in which DiI crystal was centered in the dorsomedial division of the anterior BNST. Descending projections from the dorsomedial division of the anterior BNST follow distinct trajectories to reach several hypothalamic nuclei mostly localized in periventricular zone, but not exclusively. From the dorsomedial division of the anterior BNST, DiI-positive fibers follow a periventricular route, and travel ventrally and caudally to reach the PVH, and then the ARH as described in rats (Dong and Swanson, 2006; Barbier et al., 2021). To reach the LHA, fibers can either extend from the DMH that does not display clear neuroanatomic border with the LHA, or follow the medial forebrain bundle in very lateral parts of the LHA to reach the caudal part of the brainstem (Dong and Swanson, 2006; Barbier et al., 2021).

**FIGURE 2:**
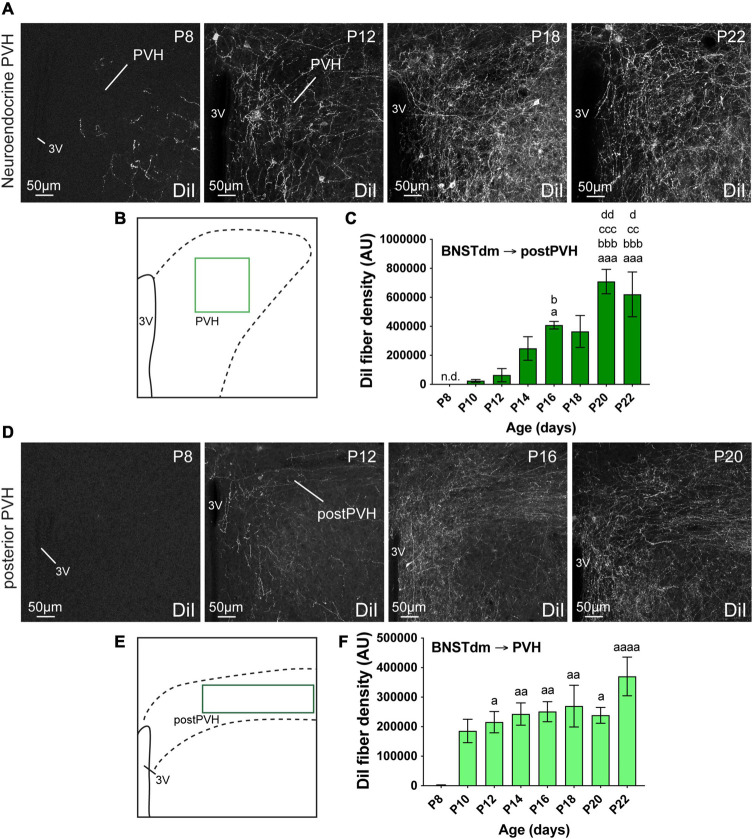
**(A)** Confocal images showing DiI-positive fibers in the neuroendocrine part of the PVH of P8, P12, P18, and P22 male mice. **(B)** Scheme illustrating the region of interest (ROI in light green, 150 × 150 μm) used to quantify the DiI-positive fiber density in the neuroendocrine part of the PVH. **(C)** Quantification of DiI-positive fiber density is summarized in the graph. **(D)** Confocal images showing DiI-positive fibers in the posterior part of the PVH of P8, P12, P16, and P20 male mice. The density of DiI-positive fibers gradually increases over postnatal ages. **(E)** Scheme illustrating the region of interest (ROI in dark green, 243 × 70 μm) used to quantify the DiI-positive fiber density in the autonomic part of the PVH. **(F)** Quantification of DiI-positive fiber density is summarized in the graph. 3V, third ventricle. Scale bars are shown in the figure. Values are shown ± SEM. Statistical significance was determined using one-way ANOVA **(C,F)**. ^*a*^*P* ≤ 0.05, ^*aa*^*P* ≤ 0.01, ^*aaa*^*P* ≤ 0.001, ^*aaaa*^*P* ≤ 0.0001 vs. P8; ^*b*^*P* ≤ 0.05, ^*bbb*^*P* ≤ 0.001 vs. P10; ^*cc*^*P* ≤ 0.01, ^*ccc*^*P* ≤ 0.001 vs. P12; ^*d*^*P* ≤ 0.05, ^*dd*^*P* ≤ 0.01 vs. P14.

**FIGURE 3:**
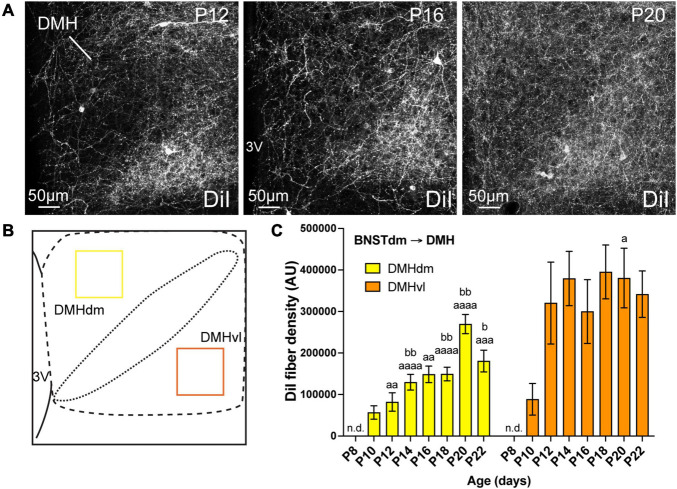
**(A)** Confocal images showing DiI-positive fibers in the DMH of P12, P16, and P20 male mice. The density of DiI-positive fibers gradually increases over postnatal ages. **(B)** Scheme illustrating the regions of interest (ROI, 115 × 115 μm) used to quantify the DiI-positive fiber density in the dorsomedial part (DMHdm, yellow) and in the ventrolateral part (DMHvl, orange) of the DMH. **(C)** Quantification of DiI-positive fiber density is summarized in the graph. fx, fornix; 3V, third ventricle. Scale bars are shown in the figure. Values are shown ± SEM. Statistical significance was determined using two-way ANOVA **(C)**. ^*a*^*P* ≤ 0.05, ^*aa*^*P* ≤ 0.01, ^*aaa*^*P* ≤ 0.001, ^*aaaa*^*P* ≤ 0.0001 vs. P8; ^*b*^*P* ≤ 0.05, ^*bb*^*P* ≤ 0.01 vs. P10.

While DiI tracer is known to mostly display anterograde diffusion, it has already been described to diffuse from terminals to cell bodies. In our samples, we observed retrogradely labeled neurons, notably in the PVH (Figure 2A), and in the ARH (Figure 3A), but this was in a very limited number of samples, and it concerned only a few neurons.

### Development of the Projections From the Dorsomedial Division of the Anterior Bed Nucleus of the Stria Terminalis to the Paraventricular Nucleus of the Hypothalamus

The PVH is composed of a rostral division that mostly contains neuroendocrine neurons, and one caudal part where the majority of intermediolateral column-projecting neurons are found (Biag et al., 2012). Both are innervated by fibers arising from the dorsomedial division of the anterior BNST (Dong and Swanson, 2006; Barbier et al., 2021). We thus assessed the ontogeny of fibers coming from the dorsomedial division of the anterior BNST by quantifying the DiI-positive fiber density in both PVH divisions (Figure 2).

Regardless the PVH subdivisions, this nucleus is among the first studied hypothalamic nuclei to receive inputs from the dorsomedial division of the anterior BNST (Figure 2). At P4, no DiI-positive fibers were observed in the PVH. At P8, only a few DiI-positive fibers were observed in the rostral part of the PVH (Figures 2A–C), while none were seen in the autonomic part at more posterior level (postPVH) (Figures 2D–F). At P10, the density of DiI-positive fiber substantially increased in the rostral part (Figure 2C, 96.7-fold-change between P8 and P10), and rapidly reached a plateau as we only observed a twofold change between P10 and P22 (Figure 2C). On the contrary, DiI-positive fiber density was gradually increasing between P10 and P20-P22 in the autonomic part of the PVH (Figure 2F, 25.6-fold change between P10 and P22).

### Development of the Projections From the Dorsomedial Division of the Anterior Bed Nucleus of the Stria Terminalis to the Dorsomedial Nucleus of the Hypothalamus

The DMH can be subdivided in dorsomedial and ventrolateral parts, and we recently showed that the ventrolateral part received greater inputs from the dorsomedial division of the anterior BNST compared to the dorsal part (Barbier et al., 2021). We quantified the DiI-positive fiber density in both dorsomedial and ventrolateral parts of the DMH (Figure 3). Like the PVH, at P4, no DiI-positive fibers were observed in the DMH but this nucleus also appears as being among the first studied hypothalamic nuclei to receive projections from the dorsomedial division of the anterior BNST (Figure 3). At P8, we did not detect fibers coming from the dorsomedial division of the anterior BNST in both studied parts of the DMH (Figure 3C). While the projections into the dorsomedial part gradually increased from P10 to P22 (Figures 3A,C, 3.2-fold change between P10 and P22), the first DiI-positive fibers were observed at P10 in the ventrolateral part and reached a plateau from P12 until P22 (Figure 3C, ranging from 88 552 AU at P10, to 320 334 AU and 341 676 AU at P12 and P22, respectively, corresponding to a 3.6-fold change between P10 and P12).

### Development of the Projections From the Dorsomedial Division of the Anterior Bed Nucleus of the Stria Terminalis to the Lateral Hypothalamic Area

**FIGURE 4:**
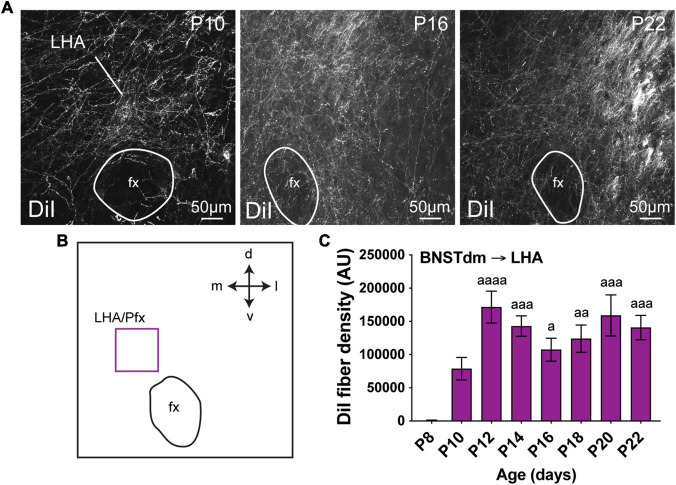
**(A)** Confocal images showing DiI-positive fibers in the LHA of P10, P16, and P22 male mice. **(B)** Scheme illustrating the regions of interest (ROI in purple, 100 × 100 μm) used to quantify the DiI-positive fiber density in the perifornical area (Pfx) of the LHA. **(C)** Quantification of DiI-positive fiber density is summarized in the graph. fx, fornix. Scale bars are shown in the figure. Values are shown ± SEM. Statistical significance was determined using one-way ANOVA **(C)**. ^*a*^*P* ≤ 0.05, ^*aa*^*P* ≤ 0.01, ^*aaa*^*P* ≤ 0.001, ^*aaaa*^*P* ≤ 0.0001 vs. P8.

LHA extends from anterior hypothalamus in PVH-containing sections until the posterior part of the tuberal hypothalamus (Franklin and Paxinos, 2008). In this study, we chose to specifically assess DiI-positive fiber density in perifornical area (at the level of the ARH and DMH corresponding to Bregma -1.7 mm in adult), as abundant fibers coming from the dorsomedial division of the anterior BNST are observed, particularly dorsally to the fornix (Dong and Swanson, 2006; Barbier et al., 2021). At P4, no fibers coming from the dorsomedial division of the anterior BNST were observed in the LHA. At P8, only a few fibers were detected in some cases, and substantially increased at P10 (Figure 4C, 106.9-fold change between P8 and P10), to reach a plateau around P12 (Figure 4C, DiI-fiber density was comprised between 171 479 AU and 140 694 AU, at P12 and P22).

### Development of the Projections From the Dorsomedial Division of the Anterior Bed Nucleus of the Stria Terminalis to the Arcuate Nucleus of the Hypothalamus

**FIGURE 5:**
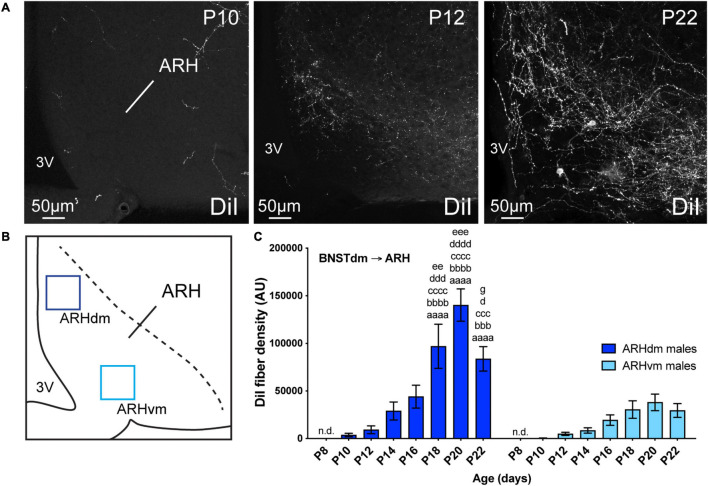
**(A)** Confocal images showing DiI-positive fibers in the ARH of P10, P12, and P22 male mice. The density of DiI-positive fibers gradually increases over postnatal ages. **(B)** Scheme illustrating the regions of interest (ROI, 90 × 90 μm) used to quantify the DiI-positive fiber density in the dorsomedial part (ARHdm, dark blue) and in the ventromedial part (ARHvm, light blue) of the ARH. **(C)** Quantification of DiI-positive fiber density is summarized in the graph. 3V, third ventricle. Scale bars are shown in the figure. Values are shown ± SEM. Statistical significance was determined using two-way ANOVA **(C)**. ^*aaaa*^*P* ≤ 0.0001 vs. P8; ^*bbb*^*P* ≤ 0.001, ^*bbbb*^*P* ≤ 0.0001 vs. P10; ^*ccc*^*P* ≤ 0.001, ^*cccc*^*P* ≤ 0.0001 vs. P12; ^*d*^*P* ≤ 0.05, ^*ddd*^*P* ≤ 0.001, ^*dddd*^*P* ≤ 0.0001 vs. P14; ^*ee*^*P* ≤ 0.01, ^*eee*^*P* ≤ 0.001 vs. P16; ^*g*^*P* ≤ 0.05 vs. P20.

**FIGURE 6:**
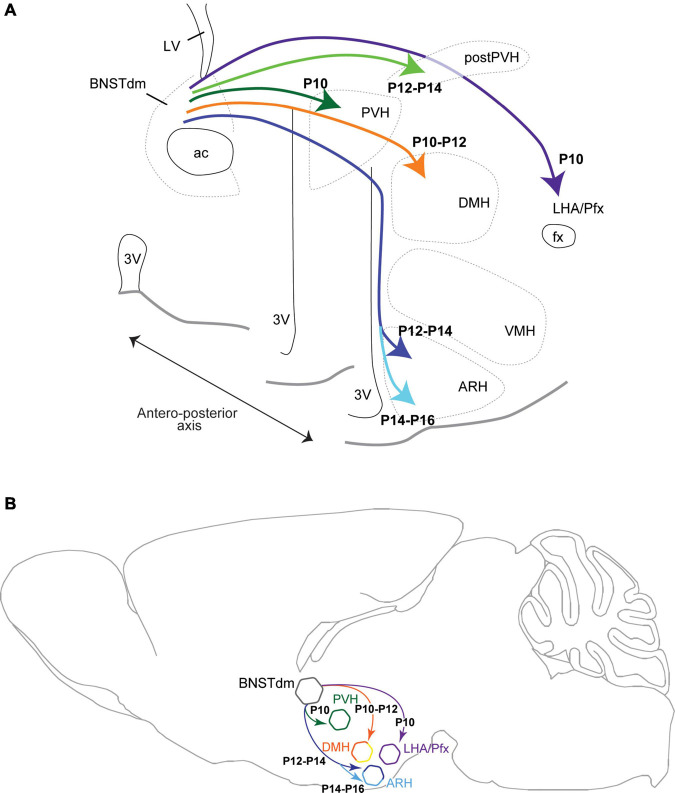
Brain coronal **(A)** and sagittal **(B)** section views showing the developmental time-course during postnatal ages of projections coming from the dorsomedial part of the anterior BNST (BNSTdm) to innervate several hypothalamic nuclei including the neuroendocrine and autonomic parts of the PVH, the DMH, the perifornical area (Pfx)/LHA and the ARH. The projections first reach dorsal parts of the hypothalamus to innervate the PVH, DMH, and LHA, to reach later the ARH by taking a periventricular and dorsal route.

Out of the studied hypothalamic nuclei, the ARH is the last nucleus that received projections from the dorsomedial part of the anterior BNST (Figures 5, 6). However, like PVH, DMH, and LHA, no DiI-positive fibers were observed at P4, as well as at P8 (Figure 5C). A 2-day delay was observed in the DiI-positive fiber detection between the dorsomedial and ventromedial part of the ARH with only a few fibers observed in the dorsomedial part at P10, and almost none in the ventromedial part of the ARH at the same age (Figures 5A,C). Between P12-P14, fibers coming from the dorsomedial part of the BNST first enter dorsally into the ARH by following a periventricular route (Figure 5A), and gradually increased until P20 to reach a postnatal peak of intensity (Figures 5A,C, 15.2-fold change between P12 and P20). At P22, the dorsomedial part of the ARH showed less DiI-positive fibers than observed at P20. This was not the case in the ventromedial division of the ARH. We used the same surface area to quantify DiI-positive fiber densities in both ARH parts (Figure 5B), and the density observed in dorsomedial part compared to ventromedial part was 2.8-fold lower at P22 (Figures 5A,C).

### Control Injections

For each crystal we implanted, we assessed the final localization, and we only used those that were DiI crystal which was restricted to the dorsomedial part of the anterior BNST for quantification. In these cases, and as we already described (Barbier et al., 2021), the VMH was devoid of DiI-positive fibers (data not shown). In some cases, the dorsomedial part of the anterior BNST was missed, and DiI crystal was observed in the adjacent septohypothalamic nucleus (ventral part of the lateral septum). In these cases, DiI-positive fibers were also observed in the PVH, ARH, DMH, and LHA, as described in rats (Risold and Swanson, 1997).

## Discussion

BNST is usually considered as a hub for the integration of stress- and anxiety-related behaviors (Walker et al., 2003; Daniel and Rainnie, 2016). Its strong connections to hypothalamic nuclei known to control goal-oriented behaviors (Dong and Swanson, 2006; Boutrel et al., 2010; Bi et al., 2012; Diniz and Bittencourt, 2017; Timper and Brüning, 2017; Smith et al., 2019; Barbier et al., 2021) revealed putative functions of these circuits in the stress-related control of feeding. Whereas we recently described the anatomy of the projections from the dorsomedial part of the anterior BNST to hypothalamic nuclei such as the PVH, DMH, ARH, and LHA (Barbier et al., 2021), there is no evidence on the developmental period in which these circuits develop. In this study, we focused our interest only in males, but we are currently assessing the sexually dimorphic aspect of these circuits using both developmental and functional approaches. Here, by using the well-described DiI-based tract tracing approach we showed that these projections, immature at birth, develop progressively during postnatal life (Figure 6) to form similar neurocircuits to that described in adult male mice. Despite DiI molecules are known to diffuse in the lipidic layer of the axonal membrane in both anterograde and retrograde direction (Makarenko, 2014), our data support the predominant anterograde property of the DiI. Indeed, in our materiel we observed intense DiI-positive fibers in the PVH, DMH, ARH, and LHA, and only a few retrogradely labeled neurons. Some studies limited the retrograde property of the DiI to the absence of reciprocal projections (Makarenko, 2014), it is clearly not the case in our study as it has been clearly established that the anterior part of the BNST receive reciprocal connections from the ARH (Bouret et al., 2004a; Betley et al., 2013; Steculorum et al., 2016), and the DMH (Thompson et al., 1996) where we observed only a few retrogradely labeled neurons.

In this study, we analyzed the DiI fiber density only every 2 days from P4 to P22, and we are aware that the described developmental time-course could present a 1-day delay/bias. Thus, out of the four studied areas, the projections from the dorsomedial division of the anterior BNST first reach the more dorsal structures of the hypothalamus including the PVH, DMH, and LHA at P10–P12. Later, the projections reach ventral hypothalamic structures following a periventricular route and enter the dorsal part of the ARH (P12–P14) to finally reach its ventral part (P14–P16). These dorsal to ventral gradient and time-course are consistent with the path followed by the projections coming from the dorsomedial part of the anterior BNST (Dong and Swanson, 2006; Barbier et al., 2021) to reach the PVH, DMH, ARH, and LHA.

In rodents, the formation of long projections has been primarily thought of as developing during embryonic life, when the brain is smaller, and local and shorter projections developing during postnatal ages. The formation of short intrahypothalamic projections is not necessarily consistent with this description as previous studies in rodents have shown that a few intrahypothalamic circuits are already developed at birth (Polston and Simerly, 2006) while others are still immature, notably the projections from the ARH to the PVH, DMH, and LHA (Bouret et al., 2004a). In our study, we showed that longer BNST to ARH projections were also immature at birth and precede the development of the reciprocal ARH to anterior BNST projections (Bouret et al., 2004a), and could serve as scaffold for the development of ARH to BNST projections by following this breadcrumb trail. Finally, other studies have shown that extrahypothalamic projections can form during embryonic life, notably the projections from the LHA to the posterior part of the brain (Croizier et al., 2011), and from the ARH to the upper thoracic spinal cord (personal data not shown). These data suggest no clear rules in the timing of the establishment of hypothalamic circuits *per se*, but could reflect the involvement of other factors such as the level of circulating hormones with leptin (Bouret et al., 2004b; Croizier et al., 2016), and ghrelin (Steculorum et al., 2015), or testosterone that directly influences the development of neuronal circuits and structures, notably the formation and differentiation of the sexually dimorphic BNST (Chung et al., 2002).

Developing environment is critical for the overall formation of neurocircuits, and this extended developmental window may increase the vulnerability of key hypothalamic circuits, and subsequently long-lasting neuronal functions. Here, all studied projections from the dorsomedial part of the anterior BNST to hypothalamic area develop when pups are on lactation as the switch from suckling to solid food occurs around P17 in mice (König and Markl, 1987). When lactating dams are fed a high fat diet, milk is enriched in insulin, leptin, glucose, and free fatty acids (Vogt et al., 2014), and the development of hypothalamic circuits is altered in offspring (Vogt et al., 2014; Park et al., 2020). Such changes in developing environment and in milk content in particular, could directly affect the expression of key guidance proteins, and subsequently the formation of hypothalamic circuits. However, while alteration of maternal metabolism and nutrition with maternal obesity, and maternal exposure to high fat diet interfere with the proper formation of the ARH to hypothalamic nuclei projections, including the PVH (Vogt et al., 2014; Park et al., 2020), there is no clear evidence on how maternal stress can impinge on the development of hypothalamic circuits and in particular, the BNST to ARH projections. Based on the literature, we can suspect that alteration of maternal environment notably maternal stress and early life stress could trigger developmental outcomes leading to metabolic dysfunctions and anxiety-like behaviors in the offspring (Delpierre et al., 2016; Derks et al., 2016; Romaní-Pérez et al., 2017). Regarding the physiological role of the anterior BNST projections to key hypothalamic circuits, deciphering how and when these circuits develop is a step forward toward a better understanding of the developmental origin of metabolic diseases.

## Data Availability Statement

The raw data supporting the conclusions of this article will be made available by the authors, without undue reservation.

## Ethics Statement

The animal study was reviewed and approved by the Veterinary Office of Canton de Vaud (#VD3193).

## Author Contributions

SC conceived the project, designed the experiments, analyzed the data, and wrote the manuscript. ML, MG, and SC performed all experiments. All authors contributed to the article and approved the submitted version.

## Conflict of Interest

The authors declare that the research was conducted in the absence of any commercial or financial relationships that could be construed as a potential conflict of interest.

## Publisher’s Note

All claims expressed in this article are solely those of the authors and do not necessarily represent those of their affiliated organizations, or those of the publisher, the editors and the reviewers. Any product that may be evaluated in this article, or claim that may be made by its manufacturer, is not guaranteed or endorsed by the publisher.
